# The effect of long-term feeding of skin barrier-fortified diets on the owner-assessed incidence of atopic dermatitis symptoms in Labrador retrievers

**DOI:** 10.1017/jns.2014.61

**Published:** 2015-02-12

**Authors:** Frank Looringh van Beeck, Adrian Watson, Margriet Bos, Vincent Biourge, Ton Willemse

**Affiliations:** 1 Department of Clinical Sciences of Companion Animals and Division of Immunology, Faculty of Veterinary Medicine, Utrecht University, Utrecht, The Netherlands; 2 Royal Canin SAS^©^, Aimargues, France; 3 Royal Canin, Veghel, The Netherlands

**Keywords:** Canine nutrition, Barrier function, Atopic dermatitis, AD, atopic dermatitis, EA, ELISA absorbance unit

## Abstract

We investigated the effect of feeding a skin barrier function-augmenting diet early in dogs' lives on the appearance of clinical signs associated with canine atopic dermatitis. Pregnant bitches (starting 5 weeks after mating) and their subsequent litters (up to 1 year of age) were fed either supplemented or unsupplemented diets. Nutrients supplemented were nicotinamide, pantothenate, histidine, inositol and choline. Circulating IgE levels to dust mute allergens Der f and Der p were measured when the puppies were 6 and 12 months old. Two owner questionnaires were used to assess the occurrence of typical signs associated with atopic dermatitis when dogs were between the ages of 22 and 36, and 34 and 48 months. Using linear mixed models we observed higher levels of circulating anti-Der f (*P* = 0·021) and -Der p IgE (*P* = 0·01) during the first year in the dogs fed the unsupplemented than in those fed the supplemented diet. The owner-assessed incidence of atopic dermatitis signs amongst the dogs was significantly greater in the unsupplemented group at the time of the second follow-up questionnaire (10/33 dogs *v.* 2/24 dogs). These outcomes suggest that a nutritionally derived improvement to barrier function early in life may reduce the frequency of signs associated with atopic dermatitis. The effect is possibly the result of making the epidermis, now thought to be a major route of environmental allergen exposure, more resistant to penetration.

Atopic dermatitis (AD) is a common skin disease of dogs defined as a genetically predisposed inflammatory and pruritic skin disorder with characteristic clinical features associated with IgE antibodies, most commonly against environmental allergens^(^^1^^)^. A diagnosis of AD is based on the animal's history and the presence of pruritus and dermatitis on specific sites of the body, such as the muzzle, ears, feet, the axillae and the abdomen. This is combined with skin test reactivity to environmental allergens or the presence of allergen-specific IgE in serum. The allergens that are commonly associated with these clinical manifestations include house dust mites, storage mites, pollen and epithelials.

Much of the research into the pathogenesis of AD in humans has focused on aberrations of the immune system that can lead to AD. Although both a defective epidermal permeability barrier and a propensity to develop secondary infections are well-recognised features of AD, it has been widely assumed that these abnormalities reflected secondary consequences of the immunological abnormalities (the historical ‘inside–outside’ view of AD pathogenesis). In more recent times, however, groups have proposed that the permeability barrier abnormalities in AD^(^^2^^)^ are not merely an epiphenomenon but rather the ‘driver’ of disease activity (‘outside–inside’ model for disease pathogenesis). For example, clinically uninvolved skin sites display barrier abnormalities such as reduced ceramide concentrations and lipid-processing enzyme activity^(^^3^^,^^4^^)^. As in humans there is increasing evidence that a skin barrier defect exists in dogs with AD. Early studies have shown abnormal intercellular stratum corneum lipid lamellae, abnormal stratum corneum morphology as well as a reduced overall concentration of skin ceramides. In addition, it has also been found that the proportion of ceramides 1 and 9 are lower in skin of atopics *v.* normal skin, along with the ratio of ceramide to cholesterol. In association with these changes, there is higher transepidermal water loss for AD skin than normal canine skin^(^^5^^–^^8^^)^. Also, the use of dog-specific lipid replacement therapy, targeting the prominent skin lipids, is seen to correct the barrier abnormalities^(^^9^^)^.

It has previously been shown that certain nutritional components when fed in combination can influence skin barrier properties as well as lipid synthesis^(^^10^^)^. These components stimulated ceramide synthesis in *in vitro* models and were subsequently shown to reduce transepidermal water loss after 9 weeks of feeding, with a further reduction after 12 weeks.

The aim of the present study was to establish if feeding a diet previously shown to improve skin barrier function could influence the occurrence of AD signs in a predisposed dog breed when fed during the early stages of life.

## Materials and methods

From 5 weeks after mating, eleven pregnant Labrador retriever dams were randomly fed either a control diet designed for pregnancy, lactation and weaning (for diet details, see Appendix A: pantothenate (7·75 mg), nicotinamide (6·75 mg), histidine (1·85 g), inositol (125 mg), choline (288 mg), pyridoxine (2·5 mg) from ingredients alone; diet B, /4184 kJ), or the same diet with further enrichment for pantothenate (50 mg), nicotinamide (163 mg), histidine (1·85 g), inositol (350 mg), choline (392 mg) and pyridoxine (20 mg) (/4184 kJ; diet A, final concentrations in diets given). EPA/DHA (1 g/4184 kJ), *n*-6 fatty acids (7·5 g/4184 kJ) and vitamin E (195 mg/4184 kJ) did not vary between the two diet groups.

A total of eighty puppies from the eleven dams' litters were subsequently enrolled into the blind, controlled study: thirty-three females and fifty-seven males. Complete litters were then fed one of the same two diets, supplemented (test diet A; *n* 35) or unsupplemented (control diet B; *n* 45), corresponding to that fed to their mother, up to 8 weeks of age. Between 8 weeks and 1 year dogs were fed a product designed for growth similarly supplemented or unsupplemented (for diet details, see Appendix A). All food batches used in the study were analysed and shown to be on target. Foods given to the parallel groups were identical apart from the supplemented ingredients, including protein sources and energy density. Breeders and owners were supplied with a single diet type to avoid cross-over mistakes and were questioned about food intake. Dog acceptance was 100 %. All diets were dry format.

Blood IgE to house dust mite allergens Der f and Der p was measured at 6 and 12 months using the Heska Allercept® test (Heska). Allercept is a non-competitive, solid-phase enzyme immunoassay that incorporates a biotinylated Fc receptor (FcεRIα) as the primary binder for allergen-specific IgE molecules, streptavidin alkaline phosphatase as the secondary layer, and *p*-ntirophenyl phosphate as substrate^(^^11^^)^. The lower threshold for a positive test is recommended by the manufacturer at 150 ELISA absorbance units (EA). A quantity of 2 ml of blood was taken from each dog, from which 1 ml plasma was prepared for the test. Tests for each time point were run in batches against calibration control samples.

Between 8 and 12 weeks of age, puppies were sold to their permanent owners. All puppies were homed individually. Owners were informed of the study, and asked to continue involvement. For their participation, they received free puppy food supplemented according to their group and provided in neutral packaging. A consent form was signed. At monthly intervals, owners were called by one of the investigators (F. L. V. B.) to ensure compliance and collect information regarding intake, palatability and digestive tolerance of the diet, as well as body weights and body condition scores. At the end of the 1-year follow-up, owners received two bags of commercial puppy food and a dog encyclopedia to reward them for their participation. At the 6- and 12-month follow-ups 2 ml blood samples were collected by breeders' regular veterinarians and sent to Utrecht University for antibody study (see above).

Following completion of the follow-up of all litters (February 2011) and once the results had been observed for the dust mite plasma antibodies, a decision was made to follow the dogs for 22 further months to assess the incidence of clinical signs compatible with AD. All questionnaires were sent out to owners at the same time (December 2011). As a consequence, at the time of this first survey the dogs were aged between 22 and 36 months. A second questionnaire was sent 1 year later at which point the dogs were between the ages of 34 and 48 months. In the questionnaires (Appendix B) the involvement of skin sites commonly associated with AD was identified. In addition, the intensity of the itch was assessed by the dog owner by means of a pruritus visual analogue scale (range 1 to 10^(^^12^^)^).

The diet fed to the dogs following completion of the 12-month controlled diet study was also recorded (as reported by the owners).

### Statistics

Statistical analyses were performed with SAS version 9·3 software (SAS Institute Inc.). A generalised linear mixed model (procGLIMIX) with Der f or Der p (< 150 EA = 0 or ≥ 150 EA =1 for both) as a binary outcome (logit transformation) was used to assess the fixed effects of time, diet, and the time × diet interaction. Dog was modelled as a random effect taking into account the longitudinal follow-up of each dog through time.

A linear mixed model (procMIXED) with Der f or Der p as a quantitative outcome was used to assess the fixed effects of time, diet, and the time × diet interaction. According to the residual distribution of each model, Der f and Der p were ranked (procRANK). Dog was modelled as a random effect taking into account the longitudinal follow-up of each dog through time. A *post hoc* 2 × 2 comparison was performed using the Schaeffer test (adjustment for multiple comparison). Results are presented as medians.

A significance level of *P* < 0·05 was used for all tests. Questionnaire data were analysed using χ^2^ to determine if signs were more common in dogs fed diet B than diet A.

### Ethics statement

All protocols adhered to European regulatory rules for animal welfare; all experimental protocols complied with European Union guidelines on animal welfare and were approved by both the Royal Canin and University of Utrecht committees for animal ethics and welfare.

Breeders participating in the study were contacted via the Dutch Labrador Club. Breeders were not paid for their involvement but all food was provided free of charge. A total of eight breeders were used, five for the group fed diet A and three for the group fed diet B.

## Results

Overall weights and growth rates for the two groups were equal up to 12 months (multivariate ANOVA; not shown). However, ANOVA per period suggested that females grew more rapidly during two monthly intervals on diet A (between the ages of 2 to 3, and 4 to 5 months), resulting in a significant weight difference between groups at 5 months. However, this weight difference disapperared between months 5 and 6.

The odds of being assigned Der f = 1 was lower at 6 months compared with 12 months (OR 0·38; 95 % CI 0·172, 0·837; *P* = 0·017). No effect of diet or interaction of diet × time was observed (*P* = 0·153 and *P* = 0·107, respectively). The odds of being assigned Der p =1 was greater for diet B compared with A (OR 0·299; 95 % CI 0·12, 0·712; *P* = 0·01). The odds of being assigned Der p = 1 was lower at 6 months compared with 12 months (OR 0·298; 95 % CI 0·133, 0·669; *P* = 0·004). No interaction of diet × time was observed (*P* = 0·584).

**Fig. 1.**
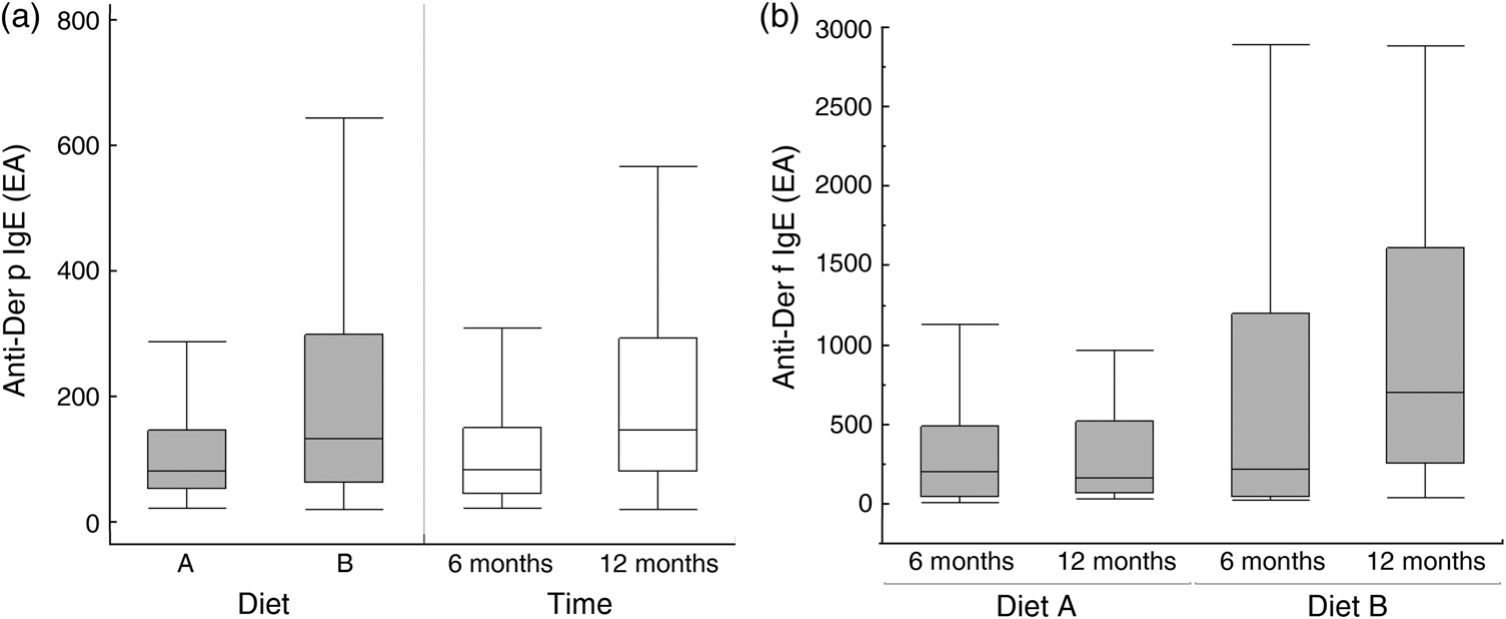
Circulating anti-Der f and -Der p IgE concentrations for two diet groups at 6 and 12 months. (a) Respective influences of diet (supplemented test diet A and unsupplemented control diet B) and time (6 and 12 months) on circulating Der p IgE (interaction NS). (b) Time effect within diet (interaction *P* < 0·001). IgE concentration is based on ELISA absorption units (EA) reported by the manufacturers of the diagnostic test. Box and whisker plots are drawn showing medians, interquartile ranges and maximum and minimum values.

For Der f treated as a quantitative outcome a significant interaction of diet × time was observed (*P* = 0·021); indeed a time effect was only observed for diet B (6 months: 214 EA *v.* 12 months: 702 EA; *P* < 0·001) and not for diet A (6 months *v.* 12 months; *P* = 0·848). For Der p treated as a quantitative outcome no interaction of diet and time was observed (*P* = 0·617). A significant effect of diet was observed (diet A: 82 EA *v.* diet B: 133 EA; *P* = 0·028). Moreover, a significant effect of time was seen (6 months: 84 EA *v.* 12 months: 146 EA; *P* < 0·001; Fig. 1).

### Questionnaire 1

The first questionnaire was sent out to the owners of all eighty Labrador puppies. After 4 weeks there were fifty-nine respondents, comprising 25/35 dogs (71·4 %) on diet A and 34/45 dogs (75·5 %) on diet B. The first follow-up questionnaire showed no difference between the two groups in the incidence of pruritus by owner assessment (Table 1). Of the dogs, three from each group showed signs of pruritus at specific locations. In the group fed diet A, two of the three dogs were from the same litter (different owners). Both of these had elevated Der f IgE at 1 year (>162 A). The third dog also had elevated Der f IgE at 1 year (192 EA). All three dogs showed clear evidence of pruritus on the abdomen and feet. Of the three dogs with pruritus in the group fed diet B, two were again from the same litter (different owners). Two of the three dogs had elevated IgE to Der f at 1 year (>508 EA).

**Table 1.** Incidence of pruritus/atopic dermatitis signs in dogs fed for their first 12 months on diet A (test diet) or diet B (control diet ) assessed by follow-up questionnaires 1 and 2 (Number of dogs in each category)

	Diet type	No pruritus	Pruritus
Follow-up 1 (age 22–36 months)†	A	22	3
	B	31	3
Follow-up 2 (age 34–48 months)*	A	22	2
	B	23	10

* Second follow-up analysis: significant difference (*P*=0·048; χ^2^ with 1 df).

† First follow-up analysis: no significant difference.

### Questionnaire 2

The second questionnaire was sent out to sixty-seven pet owners; the fifty-nine respondents to the first questionnaire and a further eight who returned their questionnaires after the deadline on the first occasion (Table 1). After 4 weeks, there were fifty-seven respondents; 24/29 diet A dogs (83 %) and 33/38 diet B dogs (87 %). Of the dogs that had been fed diet A, two demonstrated signs of AD (8·3 %). One of these dogs had been positive for pruritus signs at the first questionnaire; the other dog had developed signs during the previous year. Both dogs showed high Der f and Der p IgE at 12 months (>192 EA). Two of the originally positive dogs resolved their signs without clinical treatment or change in diet. Of the dogs that had been fed diet B, ten now showed signs of pruritus (30·3 %). Of these dogs, three also showed signs originally. Also, six of the ten had positive Der f and Der p IgE at 12 months (>205 EA); one was positive for Der f alone and another positive for Der p alone.

In the group fed diet A, the two positive dogs at the second follow-up came from the same litter. In the group fed diet B at the second follow-up, the ten positive dogs came from five different litters of the six enrolled. Of the latter, five of these dogs were from the same breeder, three from another and two from another. There was no significant difference in dog age distribution between diet groups A and B for the questionnaire phase of the study (*P* = 0·93; Kolmogorov–Smirnov test). The rates of pruritus were not significantly different between the two groups at the time of the first questionnaire (with or without the addition of the eight late responses). The rates of pruritus between the two groups were significantly different for the second questionnaire; there were more symptomatic animals in the control (B) diet group than the test diet group (A) (χ^2^; *P* = 0·048; Table 1) when all returned questionnaires were included. If the five data points are excluded where owners failed to return both questionnaires, the χ^2^ analysis returned a significance of *P* = 0·046.

No association was found between the incidence of the AD signs and dietary changes experienced by the dogs after year 1 for either follow-up questionnaire.

## Discussion

In the present study, two groups of dogs were fed two separate dietary regimens for the first year of life. The dogs were then followed via owner questionnaire at two time points until they were between 34 and 48 months of age. At the second questionnaire time point, a higher number of the group fed the control diet (B) had developed signs indicative of AD (food or non-food related) compared with the group fed the test diet containing a skin barrier supplement (group B: 30·3 % *v.* group A: 8·3 %; *P* = 0·048). Such a difference between the two sets of dogs was not seen at the time of the first owner survey (age range 22–36 months).

It has been recorded previously that pruritic signs associated with AD can be seen as early as 6 months of age^(^^13^^,^^14^^)^. However, about 75 % of dogs will show onset of clinical signs between 1 and 3 years of age. A possible reason for the later onset could be lack of exposure to the offending allergen(s), or, perhaps more likely, the requirement for a period of sensitisation following the primary exposure. In the present study, the overall increase in the number of dogs with signs associated with AD between 2 and 3 years of age could therefore be due to the time required for the population of dogs in the present study to become exposed and sensitised to environmental allergens. However, it was evident from the differential Der f and Der p IgE data between the groups that there were immunological signs of some house dust mite allergen exposure before 12 months. It is also possible therefore that the severity or regularity of pruritus signs in the affected dogs became more evident over time and so was more likely to be recorded by the second survey point. It should also be noted that there was evidence of an increased early growth rate in group A females. The likely reason was differential consumption, although none was reported by owners. It is possible that this had an impact on skin development between the groups, although the effect was temporary and disappeared within 2 months. Once permanently homed, the environments in which the dogs were maintained were not monitored. In addition, after 12 months all dogs were fed the diet of the owners' choice, which was recorded. However, no association was evident between the diet chosen and pruritus.

It was the aim of the present study to investigate the influence of a specific diet on the development of clinical signs compatible with AD and allergen-specific IgE levels in young dogs over time. The ethical committees did not feel that performing intradermal skin testing was appropriate on these ‘normal’ young dogs, as at the time of presenting they did not demonstrate overt signs of AD. A clinical diagnosis of AD is only made based on the presence of characteristic signs (diagnostic criteria according to Willemse^(^^15^^)^). The presence of allergen-specific IgE either in the skin or in serum is utilised to identify associated allergens (in the case allergen-specific immunotherapy is considered), although there is good evidence that immunotherapy outcome is not dependent on the use of either skin test or serum test results^(^^16^^–^^18^^)^. It should be noted in the present study that the serum test is only used to identify changes over time in the allergen-specific IgE serum level. As with adult dogs with AD, house dust mites are the most commonly related allergens; therefore IgE levels were measured for Der f and Der p alone. The average accuracy of the FcεRIα-based serum assay used has been calculated at 90 % (range 80–92 %, allergen dependent), the average sensitivity 86 % and average specificity 92 %^(^^19^^,^^20^^)^. It should also be noted that dogs were not assessed for all diagnostic criteria of AD, but some developed clinical signs and pruritus at sites compatible to it whereas others did not. The results showed that more of the owner-assessed signs of pruritus were observed in dogs on the control diet than the test diet.

The influence of the skin supplement on barrier function has been demonstrated previously^(^^10^^)^. There was evidence of enhanced ceramide synthesis and barrier improvement *in vitro*, and a reduction of transepidermal water loss in an *in vivo* nutritional study. The previous *in vivo* study was conducted for 12 weeks on healthy dogs; no clinical indicators were recorded as part of the present study. The study described here was designed to detect signs that feeding such a supplement during early life might have a beneficial effect on clinical parameters related to AD. That a significant difference between the groups in house dust mite allergen IgE expression, observed during the first year, preceded a significant difference in owner-observed signs of pruritus suggests that there may have been a beneficial effect of the supplement. Given the potential of the supplement to improve barrier properties^(^^10^^)^, this benefit may be a consequence of reduced ingress of allergens via the transepidermal route. Abnormalities in barrier properties associated with AD have now been well reported for both humans and dogs, even in uninvolved, apparently normal areas. It has been proposed that such abnormalities may predispose the AD sufferer to allergen exposure at the beginning of the atopic march.

The longitudinal study described here was difficult to control completely. For example, diet was not controlled beyond the first year and it was impractical to control the environment in which the animals were kept. However, the study was designed to look at the influence of early nutrition on expression of allergen IgE and subsequent owner-assessed AD signs in dogs within their normal environment. Therefore, the variability experienced by the dogs is arguably a reflection of this normality. However, it is acknowledged that the use of owner questionnaire data is not ideal, relying as it does on the subjective judgment of untrained individuals.

The ability to augment the skin barrier by providing appropriate nutrition could be a valuable tool to support an animal's natural defences against allergenic environmental agents. Subsequent studies focusing on full diagnosis and characterisation of AD in dogs fed the supplemented nutrition for a longer period will provide a clearer picture of the potential value of this approach.

## Appendix A. Diet details

### Diets A and B: early

Dehydrated poultry meat, rice, maize, animal fats, wheat gluten, poultry liver, poultry proteins, beets pulp, minerals, fish oil, soya oil, fructo-oligosaccharides, psyllium husks and seeds, l-lysine, yeast extract, taurine, egg powder, marigold extract, hydrolysed crustaceans, hydrolysed cartilage, vitamins.

Protein 30 %, fat 22 %, carbohydrate 28·1 %, N-free extract 31·5 %, metabolisable energy 4200 kcal/kg.

### Diets A and B: growth

Rice, dehydrated poultry meat, maize gluten, maize, animal fats, wheat gluten, barley, poultry proteins, hydrolysed animal protein, minerals, beet pulp, fish oil, vegetable oil (soya, borage), egg powder, fructo-oligosaccharides, psyllium husks and seeds, l-lysine, sodium polyphosphate, yeast extract, taurine, hydrolysed crustaceans, dl-methionine, hydrolysed chondroitin, l-carnitine, marigold extract.

Protein 33 %, fat 14 %, carbohydrate 31·2 %, N-free extract 36 %, metabolisable energy 3900 kcal/kg.

## Appendix B. Owner questionnaire detail

Q1. Is your dog itchy on a *regular* basis since the end of the feeding study? Yes/no*

* If ‘no’, please continue with question 6.

Q2. How many months after discontinuation of the study, the itch started:

… … … months

Q3. How severe is the itch on a scale from 1 (very minor and incidentally) – 10 (very severe and constantly): … … … . . (fill in a number)

Q4. At which age this itch started? … … … . years … … … . months

Q5. Indicate the sites at which the dog is scratching, licking, rubbing and/or biting regularly:

□ muzzle
□ ears
□ feet
□ legs
□ armpits
□ abdomen
□ elsewhere: … … …

Q6. Has your local vet diagnosed an allergy? Yes/no* (if ‘no’, please continue with question 11)

Q7. How has your vet diagnosed this allergy?

□ on the basis of clinical manifestations only
□ by means of a blood test
□ by means of a skin prick test (with different substances)
□ by means of both a blood test and a skin prick test
